# Evaluation of a laboratory-developed test for simultaneous detection of norovirus and rotavirus by real-time RT-PCR on the Panther Fusion® system

**DOI:** 10.1007/s10096-019-03697-7

**Published:** 2019-09-10

**Authors:** Robert K. Kulis-Horn, Carsten Tiemann

**Affiliations:** 1 MVZ Labor Krone GbR, Bad Salzuflen, Germany; 2 FH Bielefeld University of Applied Sciences, Bielefeld, Germany

**Keywords:** Rotavirus, Norovirus G1 and G2, Panther Fusion®, Open Access™, Laboratory-developed test (LDT), Real-time PCR, Multiplex assay

## Abstract

The Hologic Panther Fusion® Open Access™ functionality allows implementation of laboratory-developed tests (LDTs), with fully automated sample extraction, real-time PCR, and result interpretation. We report the development and validation of a multiplex LDT for norovirus G1, norovirus G2, and rotavirus from stool samples on this system. The LDT was optimized for primer and probe sequences, salt concentration, and PCR annealing temperature. Reproducibility of the PCR and extraction process was assessed. Performance of the multiplex LDT assay was evaluated with external quality assessment (EQA) samples and compared to a commercial multiplex assay (Allplex™ GI-Virus Assay, Seegene) in clinical samples. Salt concentrations and annealing/extension temperature were optimized to 4 mM MgCl_2_, 70 mM KCl, 20 mM Tris, and 60 °C, respectively. The user-prepared part of the LDT PCR mix (containing salts, probes, and primers) was stable for ≥ 11 days onboard the instrument. We observed reproducible results of PCR and the extraction process. The LDT had a sensitivity comparable to or greater than the commercial Allplex™ assay and showed excellent linearity. Forty-five EQA samples yielded the expected result with the LDT. There was 100% concordance between LDT and Allplex™ results in 160 clinical samples. Results from the suspension and direct swab stool sample preparation methods were highly concordant in the LDT. We report the successful development and validation of a multiplex PCR LDT for detection of norovirus G1, norovirus G2, and rotavirus from stool samples on the Panther Fusion® system.

**Electronic supplementary material:**

The online version of this article (10.1007/s10096-019-03697-7) contains supplementary material, which is available to authorized users.

## Introduction

Rotavirus and norovirus are highly contagious viruses that cause acute gastroenteritis leading to severe diarrhea and vomiting [1, 2]. Worldwide, diarrhea is a leading cause of life expectancy reduction in all age groups [3], accounting for approximately 1.3 million deaths in 2015, including approximately 500,000 deaths in children < 5 years old [3, 4].

Norovirus accounts for 18% of all acute gastroenteritis leading to diarrhea and vomiting [5]. Worldwide, norovirus causes 677 million cases of acute gastroenteritis in all ages, leading to approximately 214,000 deaths annually [6]. In infants and children under 5, rotavirus is the leading cause of severe diarrhea, accounting for more than 258 million episodes of diarrhea in 2016 [7]. Although rotavirus vaccination has reduced rotavirus-related death since 2004, rotavirus infections still caused approximately 128,500 deaths worldwide in children < 5 years in 2016, accounting for 29% of all diarrhea-related death in this population [7].

Six different norovirus genogroups are known and a seventh has been proposed recently [8, 9]. Norovirus infections in humans are caused predominantly by genogroup II (G2) followed by genogroup I (G1) [2, 9].

Ten different rotavirus species (A-J) have been classified on the basis of the viral protein 6 (VP6), and species A is the most common cause of rotavirus infections in humans [10, 11].

Various commercial assays are available for the detection and identification of norovirus and rotavirus in stool samples. This includes enzyme immunoassays, rapid immunochromatographic assays, and molecular tests [2, 11]. Real-time PCR assays have become the preferred method to detect gastrointestinal pathogens in stool samples due to their high sensitivity and specificity. Several real-time multiplex PCR assays for detection of multiple gastrointestinal pathogens have been developed, including the BioFire® FilmArray® Gastrointestinal Panel (BioMerieux), xTAG® Gastrointestinal Pathogen Panel (Luminex Corp.), and the Allplex™ Gastrointestinal Panel Assays (Seegene). However, these platforms are either low throughput and/or not fully automated, which is a challenge for laboratories that require high efficiencies and productivity.

The Panther Fusion® system (Hologic, Inc.) is a high throughput, fully automated, sample-to-result in vitro diagnostic (IVD) system capable of performing various IVD tests using real-time PCR, transcription-mediated amplification (TMA), or real-time TMA. Panther Fusion® has an Open Access™ functionality that allows users to perform real-time PCR laboratory-developed tests (LDT) alongside IVD tests. Laboratories can “transfer” existing LDTs or develop new LDTs on Panther Fusion®. Similar to IVD tests, LDTs developed on Panther Fusion® benefit from the system’s full automation, which encompasses all steps from sample extraction, real-time PCR, to signal detection and results interpretation.

The manufacturer provides generic RNA/DNA enzyme cartridges that contain deoxynucleotide triphosphates (dNTPs), enzymes (reverse transcriptase and DNA polymerase), and buffer needed for amplification of RNA and/or DNA templates. Components of the cartridge are in fixed concentration and lyophilized. The user-prepared primer probe reconstitution (PPR) mix is used to rehydrate the lyophilized enzymes in the cartridge. The PPR mix is composed of customized primers and probes for target and internal control (IC) detection and salt buffer in nuclease-free water. Its composition needs to be optimized for every LDT.

We took advantage of Panther Fusion®’s Open Access™ functionality to develop a multiplex LDT for the detection of norovirus G1 and G2, and rotavirus in stool samples. This is the first report of the development and validation of a multiplex LDT using Panther Fusion®’s Open Access™ functionality, including its performance in clinical samples in comparison with a commercially available reference multiplex assay.

## Material and methods

### Primer and probe design

The primer and probe sequences (Table 1) for norovirus genogroup II (G2) detection (CoG2f, CoG2r, Ring2), targeting the ORF1-ORF2 junction, were previously published [12]. We confirmed the specificity of these norovirus G2 primer and probe sequences with an alignment of 500 randomly selected norovirus G2 sequences from the National Center for Biotechnology Information (NCBI) non-redundant nucleotide collection (dataset S1).

**Table 1:** Primers and probes

Type	Name	Sequence (5′➔3′)
Norovirus G1 (primer/probe design modified from [12])		
Primer (fwd)	CoG1f-RH2	CAAGTTCCGBTGGATGCG
Primer (rev)	CoG1r-RH2	CGTCCTTAGACGCCATCATCATTTAC
Probe 1	Ring1f	FAM-TGGACAGGAGATCGCRATCTYCT-BHQ1*
Probe 2	Ring1g	FAM-TGGRCAGGAGAYCGCAATCTCCT-BHQ1*
Norovirus G2 (primer/probe design from [12])		
Primer (fwd)	CoG2f	CARGARBCNATGTTYAGRTGGATGAG
Primer (rev)	CoG2r	TCGACGCCATCTTCATTCACA
Probe	Ring2	FAM-TGGGAGGGCGATCGCAATCT-BHQ1
Rotavirus (primer/probe design modified from [14])		
Primer (fwd)	NVP3f-RH1	ACRTRACCCTCTATGAGCACA
Primer (rev)	NVP3r	GGTCACATAACGCCCCTATA
Probe	SoNVP3-RH2	ROX-AGTTAAAAGCTAACACTGTCAAAAACCTAA-BHQ2*

*fwd* forward, *rev* reverse

*Incorporation of 5-methyldeoxycytodine (underlined) in order to increase melting temperature

Primer and probe sequences for norovirus genogroup I (G1) detection were based on previously published sequences (CoG1f, CoG1r, Ring1a/b), targeting the ORF1-ORF2 junction [12]. Changes to the published sequences were made according to an alignment of 165 randomly selected norovirus G1 sequences from NCBI’s non-redundant nucleotide collection and four sequences from clinical samples. The forward primer was shifted to a more conserved region (CoG1f ➔ CoG1f-RH2). To avoid dimerization of the norovirus G2 probe (Ring2) with the norovirus G1 probes (Ring1a/b), we created a reverse complement design of the norovirus G1 probes (Ring1f/g). The length of the norovirus G1 probes was extended by several bases to increase the melting temperature. Polymorphisms present in more than 5% of the aligned norovirus G1 sequences and two additional polymorphisms detected in the sequences of clinical norovirus G1 positive samples (dataset S1) were considered in the probe design. In order to reduce probe complexity, two norovirus G1 probes were designed, each possessing two degenerate positions. Four deoxycytidines were replaced with 5-methyldeoxycytidine in order to further increase the melting temperature [13].

The primer and probe sequences for rotavirus detection were modified from previously published sequences (NVP3-Fdeg, NVP3-R1, NVP3-probe), targeting the NSP3 gene of rotavirus species A [14]. The forward primer and the probe were shifted to more conserved regions according to an alignment of 341 randomly selected rotavirus sequences (dataset S1) from NCBI’s non-redundant nucleotide collection (NVP3-Fdeg ➔ NVP3F-RH1 and NVP3-Probe ➔ SoNVP3-RH2). Only polymorphisms present in more than 5% of the aligned sequences were considered in the primer/probe design. Six deoxycytidines were replaced with 5-methyldeoxycytidine in order to further increase the melting temperature [13].

Specificity of the primers was tested in silico. A Primer-BLAST was performed with each primer set using the deposited sequences of norovirus G1, norovirus G2, rotavirus, sapovirus, adenovirus, and astrovirus as template. There was no evidence for unspecific amplification.

### Primer probe reconstitution mix

HPLC-purified, lyophilized primers and probes (Table 1) were obtained from Eurofins Genomics (Ebersberg, Germany), dissolved in nuclease-free water, and stored at − 20 °C. Nuclease-free water (DEPC treated), 1 M Tris solution (pH 8.0), 1 M MgCl_2_ solution, and 2 M KCl solution were purchased from Invitrogen/Ambion (brands of Thermo Fisher Scientific, Mass.). RNA-IC primers and probes, Panther Fusion® oil reagent, and Open Access™ primer/probe tubes and caps were provided by Hologic Inc.

For the Open Access™ functionality, a PPR mix is prepared by the user and loaded onto the instrument. The instrument uses the PPR to rehydrate a generic master mix pellet and automatically combines 20 μL of master mix with 5 μL of sample eluate to generate the final PCR mix. During optimization of the LDT, different primer, probe, and salt concentrations were evaluated. The optimal formulation of the final PCR mix is listed in Table 2.

**Table 2:** Optimal PCR mix formulation

	Concentration in final PCR reaction
KCl	70 mM
Tris	10 mM
MgCl_2_	4 mM
Primer CoG1f-RH2 and CoG1r-RH2 (each)	0.6 μM
Probes Ring1f and Ring1g (each)	0.1 μM
Primer CoG2f and CoG2r (each)	0.6 μM
Probe Ring2	0.2 μM
Primer NVP3f-RH1 and NVP3r (each)	0.6 μM
Probe SoNVP3-RH2	0.2 μM
RNA-IC fwd. and rev. primers (each)	0.75 μM
RNA-IC probe	0.5 μM

The freshly prepared PPR mix for up to 40 reactions (1200 μL) was transferred into the Open Access™ primer/probe tube, overlaid with 400 μL Panther Fusion® oil (in accordance with the manufacturer’s specifications) and spun down briefly before loading on to the Panther Fusion®.

### Sample preparation and reference assay

For Panther Fusion® Open Access™ analysis, a pea-sized amount of a clinical stool sample was suspended in 2 mL of distilled water and centrifuged (5 min, 2700×*g* at 4 °C). Three hundred twenty microliters of the centrifuged stool suspension was transferred to an Aptima® multitest swab specimen collection tube (with a penetrable cap) containing 2.9 mL Aptima® specimen transport medium (STM). This sample preparation method is referred to as “suspension” method. For comparison, we also used the “direct swab” method. A swab was directly dipped into the stool sample and transferred to an Aptima® multitest swab specimen collection tube (with a penetrable cap) containing 2.9 mL Aptima® STM. The swab remained in the tube during testing.

The Allplex™ Gastrointestinal Panel real-time RT-PCR assay (Seegene, Korea) was used as reference assay for the LDT. It detects various gastrointestinal viruses (norovirus G1, norovirus G2, rotavirus, astrovirus, adenovirus species F, and sapovirus) as well as other gastrointestinal pathogens. Sample preparation for the Allplex™ assay consisted of harvesting a pea-sized amount of a clinical stool sample, suspending it in 2 mL of distilled water, centrifuging (5 min, 2700×*g* at 4 °C), and using 50 μL of the supernatant for automated extraction in the Microlab Nimbus IVD system (Seegene) using the STARMag 96 × 4 Universal Cartridge kit according to the manufacturer’s instructions. The final elution volume was 100 μL. Five microliters of the eluate was used per reaction.

### LDT protocol

The Panther Fusion® Open Access™ feature includes the *myAccess* software to create and edit LDT protocols for the Panther Fusion®. The *myAccess* software is used to define the PCR protocol, specify target names, choose extraction reagent and detection channels, and set curve correction parameters, as well as set positivity and validity criteria. An individual norovirus/rotavirus multiplex LDT protocol was created (termed LDT-NoroRota). In this LDT, the Panther Fusion® Extraction Reagent-S was used for total nucleic acid extraction. It contains an internal RNA control that was used to monitor the extraction process, the reverse transcription, and the PCR. Sample input for extraction was 360 μL. Sample aspiration height was set to “medium” (recommended setting for samples containing particulates). Total elution volume was 50 μL. Template volume for each PCR reaction was 5 μL. The universal RNA/DNA cartridge was used. Norovirus G1 and G2 targets were analyzed in the FAM channel, rotavirus in the ROX channel, and IC in the Quasar 705 channel. Default settings of the RNA thermal profile in the *myAccess* software comprised a reverse transcription step at 46 °C (8 min), an initial denaturation at 95 °C (2 min), followed by 45 cycles of PCR comprised of 5 s denaturation at 95 °C and 22 s annealing/extension at 60 °C. Fluorescence was detected during the annealing/extension step. Real-time curve analysis parameters were modified using the *myAccess* tool and are listed in Table 3. A minimum of one positive channel (norovirus, rotavirus, or IC) was required for a valid result.

**Table 3:** LDT assay parameters

	FAM channel	ROX channel	Quasar 705 channel
Analyte	Norovirus G1 and G2	Rotavirus	IC
Analysis start cycle (cycle)	10	10	10
Baseline correction: slope limit (RFU/cycle)	250	50	25
Crosstalk correction	No	No	No
Threshold for positive result (RFU)	750	200	500
Maximum threshold cycle (*C*_T_) value	42	39	no
Minimum background fluorescence (RFU)	6000	1000	1000
Maximum background fluorescence (RFU)	14,000	3700	3700

*RFU* relative fluorescence units

### External quality assessment samples

To determine the specificity and sensitivity of the LDT, external quality assessment (EQA) samples (Instand e.V., Düsseldorf, Germany) were tested. We had access to 12 samples of the rotavirus NAT/PCR EQA scheme (EQA scheme 401; years 2015–2017) and 33 samples of the norovirus EQA scheme (EQA scheme 381; years 2014–2017). The lyophilized EQA samples were resuspended with RNase-free water according to the manufacturer’s instructions. Samples were diluted (1:10) with STM buffer for testing on the Panther Fusion® system. Genetic information was available for some EQA samples: all positive rotavirus EQA samples belonged to species A genotypes G2P[4] or G4P[8]. No further information was available on norovirus G1 strains. The genotype of seven G2 strains was known, including GII.P4, GII.P17, and three hybrid strains: GII.P16_GII.4, GII.P16_GII.2, and GII.Pe_GII.4.

### Onboard stability of the PPR mix

A PPR mix sufficient for 80 reactions was prepared and split into two tubes. Both PPR mix tubes were overlaid with oil and placed within the Panther Fusion® instrument (day 0) where they remained for the entire test period (11 days). Three clinical samples were tested (positive for norovirus G1, norovirus G2, and rotavirus, respectively). Dilutions of these 3 samples were prepared with sufficient aliquots for the entire stability test to avoid sample preparation variation on the results. Each day, each sample was extracted once and two PCR replicates were performed.

## Results

### LDT optimization on Panther Fusion®

The Open Access™ functionality allows users to optimize primer, probe, and salt concentrations in the primer probe reconstitution (PPR) mix. A representative primer/probe optimization study is described here for detection of norovirus G2: Forward and reverse primers were tested in the following concentrations: 0.2 μM, 0.4 μM, and 0.6 μM. Sensitivity improved (lower *C*_T_ and higher RFU values) with increasing primer concentrations. Therefore, primers were used at 0.6 μM for further assay development. We also tested different probe concentrations for norovirus G2 detection. With a fixed primer concentration of 0.6 μM, the probe was tested at 0.2 μM, 0.4 μM, and 0.6 μM. The maximal RFU was approximately twice as high with 0.4 and 0.6 μM probe compared to 0.2 μM. However, no change in *C*_T_ value was observed. Since the maximal fluorescence with 0.2 μM probe was sufficiently high (approx. 20,000 RFU; FAM channel), this probe concentration was chosen for further assay development.

A linear drift of the baseline was observed, which was more pronounced with higher probe concentrations. The use of probe Ring2 at 0.2 μM with probes Ring1f and Ring1g each at 0.1 μM provided a good compromise between minimal baseline drift and high sensitivity. It was possible to correct for the residual baseline drift by enabling baseline correction in the final LDT protocol (Fig. S1). No further undesired interactions between the primers and probes for norovirus G1, norovirus G2, rotavirus, and IC were observed.

With the final settings for primer and probe concentrations, a panel of nine samples was used for all optimization experiments. This panel included three different stool suspensions from clinical samples positive for norovirus G1, norovirus G2, and rotavirus, respectively (Table 4).

**Table 4:** Optimization of the PPR mix composition

		MgCl_2_ concentration (70 mM KCl, 10 mM Tris, 60 °C)			KCl concentration (4 mM MgCl_2_, 10 mM Tris, 60 °C)			Tris concentration (4 mM MgCl_2_, 70 mM KCl, 60 °C)		
No.	Target	2 mM	3 mM	4 mM	50 mM	70 mM	90 mM	5 mM	10 mM	20 mM
1	Norov. G1	38.7 ± 0.4	33.9 ± 0.3	33.4 ± 0.8	33.6 ± 0.2	35.3 ± 1.1	35.3 ± 0.2	33.5 ± 0.1	34.3 ± 0.2	34.0 ± 0.6
2	Norov. G1	33.1 ± 0.2	32.0 ± 0.2	31.8 ± 0.2	31.8 ± 0.3	32.1 ± 0.4	32.3 ± 0.6	31.8 ± 0.4	32.3 ± 0.2	32.1 ± 0.2
3	Norov. G1	28.5 ± 0.2	28.6 ± 0.4	28.1 ± 0.5	29.4 ± 0.2	29.0 ± 0.2	29.2 ± 0.5	28.7 ± 0.4	28.6 ± 0.4	28.8 ± 0.4
4	Norov. G2	38.4 ± 0.4	37.0 ± 0.5	36.1 ± 0.8	36.0 ± 0.6	35.8 ± 0.6	36.7 ± 0.4	35.3 ± 0.6	35.7 ± 0.3	35.5 ± 0.3
5	Norov. G2	35.9 ± 0.6	31.8 ± 0.2	30.5 ± 0.4	31.4 ± 0.1	31.6 ± 0.4	31.8 ± 0.2	31.6 ± 0.2	30.4 ± 0.2	32.0 ± 0.3
6	Norov. G2	29.1 ± 0.4	28.2 ± 0.2	26.0 ± 0.1	26.7 ± 0.4	27.7 ± 0.6	28.2 ± 0.6	26.8 ± 0.7	26.2 ± 0.3	28.7 ± 0.4
7	Rotavirus	32.1 ± 0.2	32.2 ± 0.3	30.9 ± 0.9	30.4 ± 0.1	30.4 ± 0.3	31.7 ± 0.4	31.1 ± 0.6	29.8 ± 0.5	32.3 ± 0.2
8	Rotavirus	30.2 ± 0.3	28.4 ± 0.5	27.7 ± 0.3	29.4 ± 0.3	27.5 ± 0.4	29.4 ± 0.9	28.5 ± 0.4	27.8 ± 0.6	28.8 ± 0.2
9	Rotavirus	30.1 ± 0.7	30.4 ± 0.2	29.7 ± 0.3	29.6 ± 0.5	29.1 ± 0.4	30.6 ± 0.3	31.1 ± 0.7	29.6 ± 0.3	29.5 ± 0.8

Each sample was extracted once and tested in three PCR replicates per condition. The table shows the mean *C*_T_ value of the three replicates ± standard deviation (SD)

MgCl_2_ concentrations of 2 mM, 3 mM, and 4 mM were tested (Table 4). The 2 mM MgCl_2_ concentration was the worst for norovirus G1 and G2 detection, yielding higher *C*_T_ values. There was no substantial difference between 3 mM and 4 mM MgCl_2_. KCl concentrations of 50 mM, 70 mM, and 90 mM were tested (Table 4). KCl concentrations had little, if any, effect on *C*_T_ values. Tris concentrations of 5 mM, 10 mM, and 20 mM were tested (Table 4). Tris concentrations had little, if any, effect on *C*_T_ values. Thus, 4 mM MgCl_2_, 70 mM KCl, and 10 mM Tris concentrations were chosen for the final LDT protocol.

**Fig. 1:**
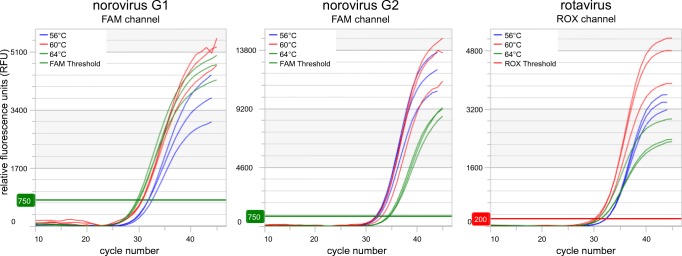
Optimization of annealing/elongation temperature. Each sample was extracted once and tested in three PCR replicates per condition. Graphs were generated with the *myAccess* software

With this final composition, three temperatures for PCR annealing/elongation were tested: 56 °C, 60 °C, and 64 °C (Fig. 1). Norovirus G1 and rotavirus detection were best at 60 °C and 64 °C, while norovirus G2 detection was best at 56 °C and 60 °C. Thus, an annealing/elongation temperature of 60 °C was well suited for the detection of all three targets and was chosen for the final LDT protocol.

### LDT reproducibility

To assess the reproducibility of the PCR reaction alone and in combination with the extraction process, the same sample was extracted three times and three PCR reactions were performed per extraction. We observed reproducible results for PCR reaction alone and in combination with the extraction process (Table 5), with a mean *C*_T_ difference < 1 cycle across replicates. The variation in *C*_T_ values was similar between the three PCR replicates of one extraction and between the different extractions, suggesting a reproducible extraction process.

**Table 5:** Reproducibility of the extraction and PCR process

No.	Target	Mean			
		Extraction 1 (*n* = 3)	Extraction 2 (*n* = 3)	Extraction 3 (*n* = 3)	All (*n* = 9)
1	Norovirus G1	33.0 ± 1.0	33.0 ± 0.1	32.1 ± 0.5	32.7 ± 0.7
2	Norovirus G1	31.3 ± 0.2	32.1 ± 0.1	31.3 ± 0.5	31.6 ± 0.5
3	Norovirus G1	27.8 ± 0.3	27.8 ± 0.5	28.3 ± 0.8	28.0 ± 0.5
4	Norovirus G2	36.3 ± 0.8	35.4 ± 0.7	35.7 ± 0.3	35.8 ± 0.7
5	Norovirus G2	30.7 ± 0.2	31.1 ± 0.4	31.3 ± 0.1	31.0 ± 0.3
6	Norovirus G2	26.3 ± 0.2	26.5 ± 0.2	27.5 ± 0.3	26.8 ± 0.6
7	Rotavirus	30.5 ± 0.9	30.9 ± 0.3	30.9 ± 0.3	30.8 ± 0.5
8	Rotavirus	29.2 ± 0.3	28.0 ± 0.4	28.5 ± 0.1	28.6 ± 0.6
9	Rotavirus	30.2 ± 0.2	29.1 ± 0.5	30.0 ± 0.2	29.8 ± 0.6

Each sample was extracted three times and three PCR reactions were performed per extraction. The table shows the mean *C*_T_ value of the three PCR replicates ± standard deviation (SD)

### Linearity and sensitivity

To assess the linearity of the assay, serial dilutions (1:10) of six clinical samples were tested (actual target concentration unknown). The samples were prepared according to the stool suspension method and tested in parallel (the same sample tube was used) with the LDT and Allplex™ reference assay to compare assay sensitivity.

**Fig. 2:**
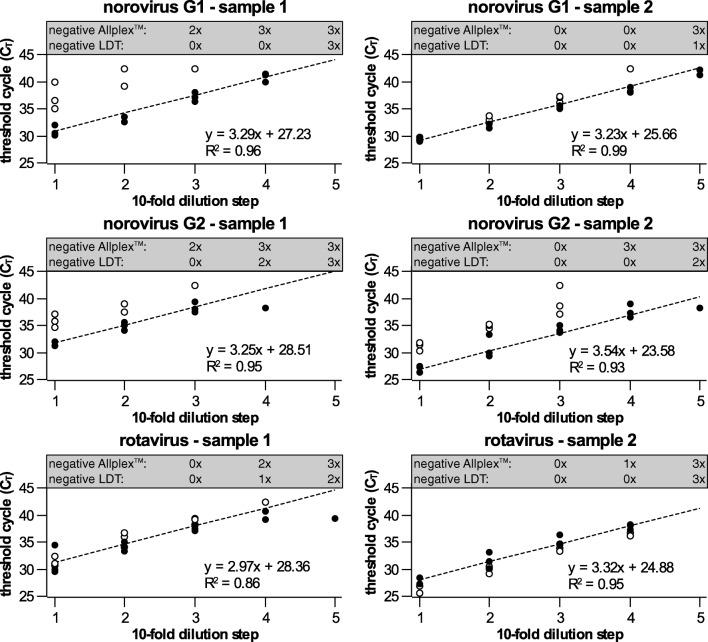
Linearity of the LDT and comparison of sensitivity to a commercial assay. Each dilution was tested in triplicate with the LDT (closed circles ●) and with the commercial Allplex™ assay (open circles ○). The dashed line indicates the linear function assuming an ideal PCR efficiency (*E* = 2). The formula represents the linear regression function for LDT results. Data for the dilution step were only included in the linear regression if at least 2 of 3 replicates were detected. The number of replicates detected as “negative” is indicated in the gray box above each graph

On average, the serial dilution resulted in an increase in *C*_T_ value by 3.3 ± 0.2 cycles, demonstrating linearity of the LDT and PCR efficiency close to the optimum (Fig. 2). Target concentrations resulting in *C*_T_ values up to 38 were reliably detected with the LDT in all replicates. *C*_T_ values > 38 were observed occasionally, but were out of the linear range. The sensitivity of rotavirus detection was comparable between the LDT and the Allplex™ assay. The sensitivity of norovirus detection was higher with the LDT, since up to two additional dilution steps were detected as positive with the LDT.

### Onboard stability of the PPR mix

**Fig. 3:**
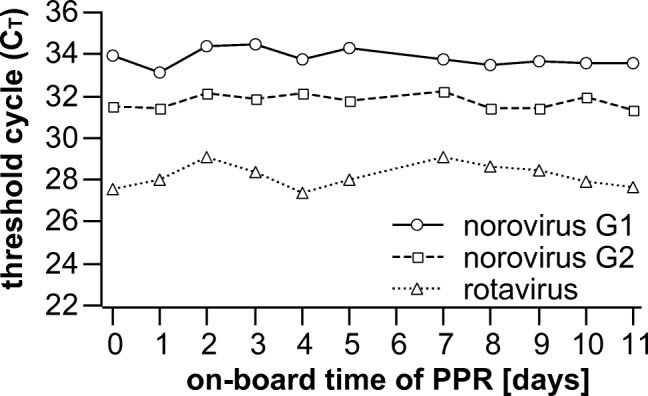
Onboard stability of the PPR mix. Three samples (norovirus G1, norovirus G2, and rotavirus) were tested every 24 h (no measurement on day 6) for a period of 11 days. Each data point represents the mean *C*_T_ value of two PCR replicates

To test the onboard stability of the PPR mix, the same three clinical samples were tested every 24 h for a period of 11 days (no testing on day 6). The *C*_T_ values remained constant for the entire test period (mean ± SD = 33.8 ± 0.4 for norovirus G1; 31.8 ± 0.5 for norovirus G2; 28.2 ± 0.6 for rotavirus) (Fig. 3). *C*_T_ values did not increase with time. These results demonstrated the stability of the PPR mix for at least 11 days.

### Evaluation of the LDT with EQA samples

To determine the specificity and sensitivity of the LDT, EQA samples for norovirus (*n* = 33; 25 positives and 8 negatives) and rotavirus (*n* = 12; 10 positives and 2 negatives) were tested.

All 10 positive rotavirus EQA samples and all 25 positive norovirus EQA samples tested positive for the respective virus with the LDT. All negative rotavirus and norovirus EQA samples tested negative with the LDT.

### Evaluation of the LDT with clinical samples

To investigate the sensitivity and specificity of the LDT, 160 clinical stool samples previously tested with the Allplex™ assay were retested with the LDT. These 160 samples consisted of 25 samples positive for rotavirus, 25 samples positive for norovirus G1, 50 samples positive for norovirus G2, and 60 samples negative for both viruses. Of these 60 negative samples, 10 were positive for adenovirus species F, astrovirus, or sapovirus, respectively.

There was 100% concordance between LDT and Allplex™ results. All clinical samples that previously tested positive for rotavirus or norovirus in the Allplex™ assay also tested positive for the respective virus in the LDT. All samples that were previously tested negative for rotavirus or norovirus were also negative in the LDT. In particular, all samples positive for adenovirus species F, astrovirus, or sapovirus were negative for noro- and rotavirus in the LDT, demonstrating the high specificity of the LDT.

### Comparison of stool sample preparation methods

Two stool sample preparation methods were compared: the suspension method and the direct swab method (see “Materials and Methods”). Fifty-nine stool samples with known status by the Allplex™ assay were tested: 30 samples positive for norovirus G1, norovirus G2, or rotavirus (including two norovirus/rotavirus double infections), and 29 samples negative for all three pathogens. These samples were not retested with the Allplex™ assay.

There was a good concordance of results from the two stool sample preparation methods (Table 6). There was no issue (i.e., PCR inhibition) caused by solid stool particles present on the bottom of the collection tubes when using the direct swab method. The direct swab method yielded a higher sensitivity. Three samples that previously tested positive for rotavirus (1 sample) or norovirus (2 samples) were positive only with the direct swab method (i.e., were missed by the suspension method) in the retest. *C*_T_ values of these three samples were rather high in the Allplex™ assay and with direct swab testing, suggesting a low viral load (data not shown). On average, *C*_T_ values were lower by 2–4 cycles with the direct swab method (data not shown), demonstrating the advantage of this sample preparation method with respect to sensitivity. The unexpected norovirus detection in one of the astrovirus positive samples (*C*_T_ = 40.1) is most likely explained by a low viral load of the sample that was not detected with the Allplex™ assay.

**Table 6:** Comparison of the suspension and direct swab stool sample preparation methods

Previous result (Allplex™; Seegene)	Positive LDT result rotavirus		Positive LDT result norovirus	
	Suspension	Direct swab	Suspension	Direct swab
Positive samples				
Rotavirus (*n* = 9)	8	9	0	0
Rota- and norovirus G1 (*n* = 2)	2	2	1	2
Norovirus G1 (*n* = 9)	0	0	8	9
Norovirus G2 (*n* = 10)	0	0	10	10
Negative samples (for rotavirus and norovirus)				
Adenovirus F positive (*n* = 5)	0	0	0	0
Astrovirus positive (*n* = 4)	0	0	0	1
Sapovirus positive (*n* = 5)	0	0	0	0
Negative for all viruses (*n* = 15)	0	0	0	0

## Discussion

Herein, we demonstrated the successful development and validation of a multiplex real-time RT-PCR LDT for detection of norovirus G1, norovirus G2, and rotavirus from stool samples on the Panther Fusion® using its Open Access™ functionality. Various commercial IVD tests are available on the Panther Fusion® system (e.g., for the detection of respiratory pathogens [15]), but this is the first report of an LDT that takes advantage of the fully automated Panther Fusion system, including sample extraction, real-time PCR reaction, and result interpretation with a bidirectional laboratory information system (LIS) interface.

Six parameters are open for optimization in the LDT: (i) primers (sequence and concentration), (ii) probes (sequence and concentration), (iii) MgCl_2_ concentration, (iv) KCl concentration, (v) Tris concentration, and (vi) annealing/elongation temperature. Adequate selection of primer and probe sequences is key to yield high assay sensitivity and specificity. Different primer and probe sequences for real-time RT-PCR detection of norovirus and rotavirus have been published. With norovirus G2 detection, already published hydrolysis probe protocols designed for a different real-time RT-PCR instrument [12] could be easily transferred to the Panther Fusion® system without the need for extensive optimization. Changes to the published primer and probe sequences for norovirus G1 [12] and rotavirus [14] detection were primarily made to allow for multiplexing and to cover more variants.

MgCl_2_, KCl, and Tris concentrations can be easily changed in the LDT. However, there was no need for extensive optimization. MgCl_2_ concentration was the most impactful (lower sensitivities observed with lower MgCl_2_ concentrations), while KCl and Tris concentrations had no effect on sensitivity at recommended ranges. Thus, salt concentrations of 4 mM MgCl_2_, 70 mM KCl, and 20 mM Tris worked well in the LDT. The annealing/elongation temperature can be adjusted from 55 to 65 °C. As expected, the annealing/elongation temperature had an important impact on LDT sensitivity.

Using the Panther Fusion® Extraction Reagent-S in the LDT, we observed a reliable norovirus and rotavirus RNA extraction process from stool suspensions with no need for further optimization. The direct swab method for stool sample preparation showed greater sensitivity than the suspension method and is a convenient method that reduces hands-on time for sample preparation. The most important components for the reverse transcription and PCR reactions, including dNTPs, enzymes, and buffer components, are provided in a ready-to-use RNA/DNA enzyme cartridge in a lyophilized form. From our experience, the composition of the universal RNA/DNA cartridge is adequate, resulting in satisfactory and reproducible reverse transcription and PCR results.

Several LDT results processing features are available in the Panther Fusion® software, including curve correction, various criteria for positivity, and channel and sample validity. Baseline correction was used to correct for the linear, reproducible, and non-target-specific drift of baseline in the FAM channel, which was mainly caused by the combination of the three probes for norovirus G1 and G2 detection. The use of baseline correction eliminated false-positive results and enabled more reproducible *C*_T_ values without the need for probe redesign. There was only a minimal baseline drift in the ROX (rotavirus) and the Quasar 705 (IC) channels. Crosstalk correction was unnecessary, since no channel-to-channel bleed from the fluorescence signals were observed. Crosstalk correction may be needed if additional dyes are used. The Open Access™ functionality has five available fluorescence channels available. Our LDT only uses three of these channels (FAM, ROX, and Quasar 705 for norovirus, rotavirus, and IC, respectively). If needed, differentiating between norovirus G1 and G2 infections could be easily achieved by changing the fluorophore of the norovirus G2 probe from the FAM to HEX channel. One could also add additional targets (e.g., adenovirus or sapovirus) using the two unused channels.

Positivity criteria were defined using *C*_T_ threshold and maximum *C*_T_. The *C*_T_ threshold can be defined for each channel individually. The *C*_T_ threshold should be high enough to distinguish true-positives from unspecific background, but not too high to miss low-positive samples. Additionally, mismatches in primer/probe sequences lead to reduced signal intensity; this phenomenon was observed with various norovirus G1 samples during our study (data not shown) and is due to its high genetic variability [8, 12] in the primer/probe target region. Therefore, the FAM *C*_T_ threshold was set to a rather low value of 750 RFU to reliably detect all norovirus G1 variants. The maximum *C*_T_ value was set to 42 for norovirus and 39 for rotavirus detection. Samples with *C*_T_ values greater than the maximum *C*_T_ were considered negative, even if they generated a signal. This rule was established for two reasons: (i) clinical significance and (ii) potential cross-contamination during stool sample preparation. Usually, acute norovirus or rotavirus infections yield high viral loads within the stool sample, resulting in low *C*_T_ values (< 20). Positive results with high *C*_T_ values (> 35) may have little or no clinical relevance, suggesting a cleared infection, as virus shedding can continue for several days after symptoms have subsided [11, 16]. These high viral loads during an acute infection also hold a higher risk of cross-contamination during stool sample preparation. No evidence of cross-contamination within the instrument was observed during our study. The maximum *C*_T_ setting reduces the risk of reporting false-positives caused by cross-contamination and should only obscure few true-positives, if any, with an uncertain clinical relevance.

Different additional sample validity criteria can be defined for LDTs in the *myAccess* software. These criteria allow for discrimination between true-negative results and false-negative results caused by technical failure or RT-PCR inhibitors. In the present LDT, at least one positive signal in any of the three channels used was required for a valid result. In the case of very high target concentrations in the sample, the IC may not be amplified due to competition effects. However, in our experience, the IC was reliably amplified even with high target concentrations, with no major changes in *C*_T_. Therefore, a mandatory signal in the IC channel may also be applied.

The multiplex LDT showed a very good sensitivity and specificity. The LDT was at least as sensitive in the detection of both viruses as a CE-cleared reference assay, which claims to have a limit of detection (LoD) of 75 tissue-culture infectious dose 50 (TCID_50_) units per milliliter sample for norovirus G1, 5 TCID_50_/ml for norovirus G2, and 50 RNA copies per reaction for rotavirus according to the manufacturer (user manual Allplex™ GI-virus assay 05/2017 V2.0), and that has demonstrated excellent agreement with other molecular assays for stool pathogen diagnostics [17].

No discrepant results were observed when testing 160 clinical samples with the LDT and the Allplex™ assay in parallel. No cross-reaction with other common stool viruses causing diarrhea (adenovirus F, astrovirus, sapovirus) was observed. The LDT also generated the expected results with all EQA samples tested.

## Conclusion

The Panther Fusion® system has a fully automated workflow with minimal hands-on time. Herein, we demonstrate that it is easily possible to implement LDTs on this system, using the detection of norovirus and rotavirus from clinical stool samples as example. The sample extraction, PCR set-up, PCR reaction, and result interpretation are performed in one step without manual intervention. The user only has to prepare the stool samples and the PPR mix. New samples can be loaded at any time thanks to random continuous access. The time to results can be as short as 2 h and 30 min. Samples are processed five at a time, with results for 5 samples coming out every 5 min. The system can be bidirectionally connected to the LIS to receive sample information and send results, without the need of error-prone manual transfer. Our noro-/rotavirus multiplex LDT performed comparably or better in respect to specificity and specificity than a commercial, CE-cleared reference assay, allowing for fast and reliable diagnostics in clinical routine.

## Electronic supplementary material

Fig. S1 (PDF 202 kb)

ESM 1 (XLSX 820 kb)
