# Supplementary material for: Evaluation of a laboratory-developed test for simultaneous detection of norovirus and rotavirus by real-time RT-PCR on the Panther Fusion® system
Source: Eur J Clin Microbiol Infect Dis. 2019 Sep 10;39(1):103–12. doi: 10.1007/s10096-019-03697-7 (PMC6962121; doi:10.1007/s10096-019-03697-7)
Supplement: Supplementary file 1 — (PDF 202 kb) [file 10096_2019_3697_MOESM1_ESM.pdf]

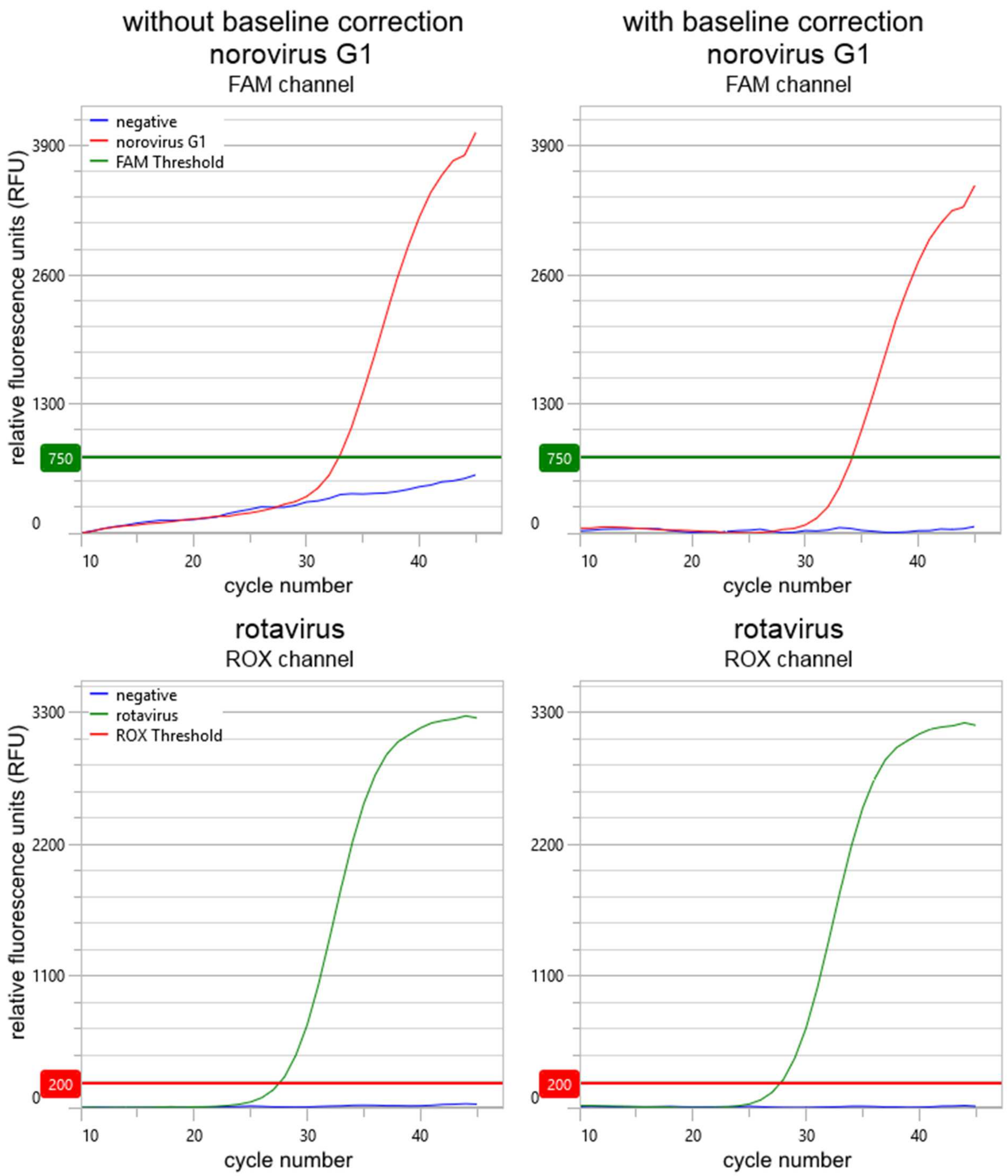

Fig. S1: Effect of baseline correction on curve shape in the FAM and ROX channel. Graphs were generated with the *myAccess* software. Baseline correction slope limit was set to 250 RFU/cycle in the FAM channel and 50 RFU/cycle in the ROX channel.
